# Digital Health in Response to COVID‐19 in Low‐ and Middle‐income Countries: Opportunities and Challenges

**DOI:** 10.1111/1758-5899.12880

**Published:** 2021-03-17

**Authors:** Elizabeth A. Mitgang, Joaquin A. Blaya, Mickey Chopra

**Affiliations:** ^1^ World Bank

## Abstract

COVID‐19 has pulled back the curtain on health system fragility to expose persistent and deepening inequities worldwide. The limited capacity of low‐ and lower‐middle income countries (LMICs) to respond to the pandemic and its impact on the health of populations – particularly the most vulnerable – presents a marked challenge. In this context, countries face the enormous task of rethinking the way essential services will be delivered. A critical and essential part of solving these challenges will be using information and communication technology and digital health to enhance direct communication with the public; scale proven and innovative service delivery models; and empower the frontlines. However, if the deployment, adaptation, or expansion of these innovations are not user‐centered for the most marginalized or do not learn from past lessons, it could be highly wasteful at best. At worst, such shortcomings could exacerbate pre‐existing weaknesses in the health care system such as exclusion of peripheral populations, disempowerment of health workers, and proliferation of unregulated private providers. We provide recommendations of which innovations should be prioritized and implementation principles to address the current challenges while responding to the need to fundamentally change service delivery for accelerated impact.

## Abstract 2

While state of the pandemic remains urgent and overwhelming, novel and practical service delivery models can be implemented at‐scale and designed for the needs of populations who need them most.

The novel coronavirus, COVID‐19, has pulled back the curtain on health system fragility exposing persistent and deep inequities. Across LMICs, approximately 8.6 million people succumb to causes amenable to routine health care provision annually (Kruk et al., 2018). Now, these same systems are in the precarious situation of maintaining routine service delivery – such as reproductive, maternal, newborn, child health and nutrition (RMNCHN) (Abbas et al., 2020; Roberton et al., 2020), malaria (World Health Organization, 2020a), tuberculosis (Cilloni et al., 2020), and HIV (Hogan et al., 2020) programs – while concurrently responding to this unprecedented, exogenous shock. Compounding this situation is a lack of applicable mitigation measures for the least well‐off, including those residing in slums, displaced migrant communities, and deep rural geographies. In this context, fundamentally rethinking service delivery to ensure the well‐being of frontline health workers – those central to sustaining these systems – as well as the populations they serve through to the last mile is essential. Information communication technology (ICT) and digital health figure prominently into this process.

Multiple factors contribute to the promotion of ICT and digital health as key enablers of service delivery innovation. These include the expansion of mobile phone penetration and internet access; national digital health strategies and comprehensive policies; growth of commercial ICT markets; and a proliferation of resources on technology selection, implementation guidance, and evidence of impact (World Health Organization, 2012; PATH, 2019; UNICEF, 2016). In addition to augmenting service delivery models that currently exist in health systems, strategic implementation of new innovations can also play a role in bypassing conventional bottlenecks or weaknesses. Here we highlight some ways by which tech‐enabling service delivery has the potential to both combat COVID‐19 in the near term and drive systems toward the longer‐term goal of universal health coverage.

## Enhancing direct communication with the public

Basic communication messaging platforms, social media, and chatbots can serve as a first line of defense against misinformation, which often spreads at a viral rate. By making a concerted effort to enhance communication, governments and health authorities can rapidly disseminate authenticated information and address health inquiries in the appropriate language and cultural context. In Malawi, VillageReach and the Ministry of Health leverage a national health information line, Chipatala cha pa Foni (CCPF) or Health Center by Phone. In less than a decade, CCPF has grown from providing RMNCHN services in a single district to scaling nationally and addressing all health topics with a quick pivot to include COVID‐19. Through the pandemic, the government has endorsed the national hotline as a reliable source for COVID‐19 information. Beyond Malawi, Viamo’s 3‐2‐1 interactive voice response (IVR) hotline has reached over 14 million listeners through upwards of 43 million message engagements across more than 25 countries to date worldwide – including Haiti, Nepal and Pakistan – through its COVID‐19 response (Viamo, 2020).

The World Health Organization (WHO) has developed a communication platform through WhatsApp to disseminate trusted information directly to diverse populations globally in response to the pandemic. The WHO’s Health Alert service allows users to message ‘hi’ in one of seven languages, prompting a dialogue across a range of COVID‐19 topics – from symptoms to situation reports – enabled by chatbot technology. By partnering with a messaging service already embedded in the daily lives of citizens around the world, Health Alert can connect with as many as 2 billion people (World Health Organization, 2020b).

## Scaling proven, innovative service delivery models

Innovative models for service delivery often use digital health to help lower the barriers to receiving care. The most prominent of these is telemedicine, which allows health workers to connect with patients remotely so that access does not jeopardize safety. Telemedicine not only reduces in‐person patient visits, it also maximizes human resources. For example, Babyl Rwanda provides virtual triage and primary care services via mobile phone with post‐consultation prescription or lab test codes sent by text message redeemable at public and private facilities. With over one million consultations completed since its launch in 2016 and 75 per cent of medically motivated consultations classified as digitally treatable, the technology was primed for pandemic response (Dalberg, 2019). The platform now helps keep patients out of facilities while complementing a government call center and automated symptom checker via feature codes. Another such example is Halodoc, which provides users with access to live video consultations with more than 20,000 licensed doctors in the country through mobile app and desktop platforms. Reaching 2 million users across Indonesia, Halodoc has also partnered with 1,200 pharmacies across 30 cities in Indonesia and Go‐Jek, a driver‐on‐demand service, to deliver medicine directly to patients (Mobihealth News, 2019). More recently, the platform has evolved to include appointment scheduling for COVID‐19 testing among participating hospitals.

## Empowering the frontlines

Task shifting from primary health facilities to community health workers (CHWs) is another way to reimagine the delivery and maintenance of essential services while unburdening health facilities. Globally, hundreds of thousands of CHWs already use smartphones and applications such as CommCare, Community Health Toolkit (Medic Mobile), and OpenSRP. In such settings, these applications can be rapidly updated with forms and automated protocols to assist with the pandemic response and perform tasks including triaging and referring suspected COVID‐19 cases, recording vital patient information, and building trust with community members through hygiene counseling and de‐stigmatization. By fortifying these community linkages, CHWs can play an integral role in reducing the need for patients to visit health facilities.

Additionally, virtual peer‐to‐peer learning and tele‐mentoring platforms can facilitate the dissemination of new skills and information, particularly when the landscape of available guidance is changing in real‐time. One example is Project ECHO. This platform leverages videoconferencing to connect communities of practice virtually including, but not limited to, networks addressing COVID‐19 treatment and clinical operations. Over 800 ECHO programs exist across nearly 40 countries including India, Brazil and South Africa. As early as February 2020, Project ECHO co‐hosted specialized COVID‐19 virtual learning sessions connecting nearly 2,000 health providers in 20 (largely LMIC) countries across 400 sites (Project ECHO, 2020).

The above examples highlight the penetration and potential of technology even in the poorest countries. However, these same innovations can also risk further exacerbating existing health system weaknesses including the exclusion of marginalized populations; the disempowerment of health workers; and the proliferation of unregulated data sharing and private providers. At the most fundamental level, not everyone has equitable access to digital health technology. Many individual users will not have a way to overcome the salient barriers preventing inclusive use – a divide too often characterized by socioeconomic, geographic, and gender disparities (Bahia, 2019).

When available, digital health tools should be designed for the people they serve and for the environments in which they will be used (Waugaman, 2017). For example, too often mobile health (mHealth) applications rely on consistent or costly internet connectivity. This reality diminishes the utility of the innovation for extreme users like CHWs serving remote populations or humanitarian workers serving fragile, conflict, and violence settings. The appropriate digital health environment is also enabled by effective policy and regulation. Across Africa, over 75 per cent of countries have developed national digital health strategies and architectures (Vota, 2019); however, many of these countries do not articulate or execute clear policies for data security and protocols for electronic data exchange. This poses particular risk in countries with a pronounced, unregulated private technology sector where the risk of commodification and dissemination of unconsented user data is heightened (Tiffin et al., 2019). Taking the greater digital ecosystem into consideration can help ensure that we solve service delivery challenges in context, rather than implement something that lacks scalability, interoperability, or privacy.

While the state of the pandemic remains urgent and overwhelming, novel and practical service delivery models can be implemented at‐scale and designed for the needs of populations who need them most. In taking these innovations to scale, we must remain acutely aware of inherent health system inequities and weaknesses that stand to worsen as a result of poor planning and execution. By assessing the entirety of the system, understanding the needs of end‐users, and effectively leveraging the appropriate building blocks (e.g. robust community health work forces, high mobile phone penetration, and enabling regulatory environments) countries are favorably positioned to leapfrog traditional brick‐and‐mortar facilities that often lack efficient and sustainable service delivery and equitable health outcomes. With the uptake of new and improving technology diffusing across LMICs, the opportunity to reimagine service delivery through digital health innovation is real, not just theoretical.
